# Temperature and precipitation differently influence entomological indices based on larval or adult sampling

**DOI:** 10.1111/mve.70099

**Published:** 2026-07-28

**Authors:** Angie Nataly Aire Laureano, André de Souza Leandro, Rafael Maciel‐de‐Freitas, Wagner Antonio Chiba‐de‐Castro

**Affiliations:** ^1^ Latin American Institute of Life and Natural Sciences Federal University for Latin American Integration (UNILA) Foz do Iguaçu Paraná Brazil; ^2^ Centro de Controle de Zoonoses Secretaria Municipal de Saúde de Foz do Iguaçu Foz do Iguaçu Paraná Brazil; ^3^ Laboratório de Mosquitos Transmissores de Hematozoários Instituto Oswaldo Cruz, Fiocruz Rio de Janeiro Rio de Janeiro Brazil; ^4^ Department of Arbovirology and Entomology Bernhard Nocht Institute for Tropical Medicine Hamburg Germany

**Keywords:** *Aedes aegypti*, arboviral disease, climate change, entomological indices, LIRAa, public health

## Abstract

Dengue is an arboviral disease widely distributed in the world, with high incidence in tropical and subtropical regions. The population dynamics of *Aedes aegypti* (Diptera: Culicidae), the main vector of the virus, are strongly influenced by climatic variables that modulate the occurrence and intensity of infestations. This study evaluated the influence of temperature and precipitation on the presence of the vector, integrating entomological and meteorological data. Larval and adult indicators obtained through Rapid Assessment of Larva Index (LIRAa) and Adultrap traps were used, analysed by generalized linear mixed models (GLMMs) and Akaike information criterion (AIC) in the municipality of Foz do Iguaçu, Brazil. The results demonstrated a significant association between larval indices (House Infestation Index [HI] and Breteau Index [BI]) and the climatic variables, indicating that the increase in temperature and precipitation favours mosquito infestation. In contrast, the adult indices (Trap Positivity Index [TPI] and Adult Density Index [ADI]) did not show a statistically significant relationship, suggesting behavioural adjustments of the vector in face of environmental conditions. The HI model indicated temperature as the determining factor in the presence of immature stages. These findings reinforce the usefulness of entomological monitoring, highlighting its importance in integrated vector‐control strategies that consider climatic seasonality, especially thermal variation.

## Graphical abstract

- Larval indices, especially house infestation index (HI) and breteau index (BI), were significantly associated with temperature and precipitation, indicating that warmer and wetter conditions increase *Aedes aegypti* breeding‐site positivity.
- Adult indices, trap positivity index (TPI) and adult density index (ADI) showed no significant association with climatic variables, suggesting adult mosquito dynamics are shaped by microhabitats, behaviour and operational factors.
- The HI was the strongest climate‐sensitive indicator, with mean temperature providing the best‐supported model for predicting immature *A. aegypti* infestation.

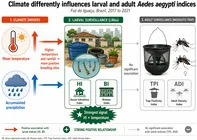

## INTRODUCTION

Dengue fever is a zoonotic viral disease that has become a major global public health concern due to the notable increase of human cases in the last decades (Guzman et al., 2010; Guzman et al., 2016). An estimated 5.6 billion people live in areas under dengue transmission risk, resulting in 100 to 400 million dengue cases annually, making it the fastest‐spreading mosquito‐borne disease worldwide (WHO, 2025). In most of the endemic areas, transmission occurs through the bite of the female *Aedes aegypti* mosquito, the principal vector of the four dengue virus serotypes. Other mosquito species such as *A. albopictus* and *A. polynesiensis* have local importance, especially in the absence of the primary vector *A. aegypti* (Ahebwa et al., 2023; Calvez et al., 2020; Oxborough et al., 2025). In 2024 alone, Latin America and the Caribbean faced the largest dengue epidemic ever recorded, with more than 10 million reported cases and 5000 deaths (Gurgel‐Gonçalves et al., 2024). This marked increase is directly linked to the biology of *A. aegypti* and to environmental factors intrinsic to the region, such as unplanned urban expansion and a favourable climate, allowing colonisation of new areas that were considered free of *A. aegypti* infestation in the recent past (Ali & Ahmad, 2019; Lee et al., 2021).

Temperature and precipitation directly modulate mosquito population growth and are considered key determinants of vector ecology and, consequently, dengue virus transmission (Dennington et al., 2025; Mordecai et al., 2019; Stewart Ibarra et al., 2013). Oviposition generally occurs on the moist surfaces near stagnant water of breeding sites with low organic material, such as water tanks, tyres and metal drums (Maciel‐de‐Freitas et al., 2007), with eggs hatching once submerged, that is, typically during rainfall events. Egg development accelerates at temperatures above 25°C, with the larval stage lasting 5–7 days and the pupal stage approximately 2 days. Adult mosquito longevity ranges 2–4 weeks, though it can be influenced by extrinsic factors such as interspecific competition with other mosquito species and elevated temperatures (Valle et al., 2015). Elevated temperatures are therefore associated with shorter life cycles, increased vector activity and enhanced phenotypic variability across environments (Sippy et al., 2019). Precipitation, in turn, plays a more complex role: while moderate rainfall may create suitable oviposition sites, heavy rainfall can destroy immature stages through flushing events (Koenraadt & Harrington, 2008).

The population dynamics of *A. aegypti* exhibit significant spatiotemporal variation, shaped by microclimatic conditions and strongly affected by seasonal fluctuations and habitat availability. These dynamics hinder outbreak prediction and undermine the effectiveness of control interventions in highly endemic areas such as metropolitan areas in the tropics (Leandro, Pires‐Vieira, et al., 2024; Reiner et al., 2019). Multiple environmental factors form a complex network of interactions that govern the spread of *A. aegypti* and the diseases it transmits (Hales et al., 2002). Among these, temperature and precipitation stand out as primary modulators of population dynamics, creating conditions favourable to vector reproduction and dispersal. In tropical and subtropical regions, the combination of high temperatures and moderate precipitation typically intensifies mosquito population growth, resulting in seasonal dengue outbreaks (Gurgel‐Gonçalves et al., 2024). Climate change is expected to further alter these infestation patterns, expanding vector distribution into areas previously unsuitable for its development due to rising mean temperatures (Moser et al., 2023). This scenario underscores the need for integrated vector‐control strategies that explicitly consider seasonal climatic variability, particularly temperature and precipitation, to enable more effective intervention planning.

In Brazil, *A. aegypti* control efforts rely on entomological surveillance tools designed to assess mosquito distribution and abundance. The Rapid Index Survey for *A. aegypti* (LIRAa) is widely used to monitor vector infestation in urban areas. It consists of the systematic inspection of around 5% of city households to identify larval breeding sites, enabling the calculation of indicators such as the House Infestation Index (HI) and the Breteau Index (BI), which guide targeted control actions in high‐risk areas (Coelho et al., 2008). Adult mosquito traps, such as the Adultrap, have also shown strong effectiveness for monitoring. Designed to capture adult female vectors, these traps offer a more direct estimate of the transmitting population (Maciel‐de‐Freitas et al., 2008). In addition to their high sensitivity, they allow for subsequent viral testing of captured mosquitoes, making them a promising strategy for integrated surveillance and helping to overcome limitations associated with methods that focus exclusively on immature forms (Codeço et al., 2015; Leandro, Ayala, et al., 2022; Leandro, de Castro, et al., 2022; Leandro, Pires‐Vieira, et al., 2024).

This study aims to analyse the influence of climate on *A. aegypti* populations in the high‐endemic city of Foz do Iguaçu, Paraná. To achieve this, we compare historical temperature and precipitation data from the municipality with entomological monitoring indicators derived from both LIRAa (larval surveys) and Adultrap (adult mosquito captures). We hypothesise that higher accumulated precipitation and increased mean temperature are associated with an increase in breeding sites positivity and higher occurrence of adult mosquitoes.

## METHODS

### Study area

The present study was conducted in the municipality of Foz do Iguaçu (25°30′58″ S, 54°35′07″ W), located in the Triple Border region shared by Brazil, Argentina and Paraguay. The municipality comprises 73 urban areas, each containing approximately 1500 properties, including ground‐level residential and commercial establishments, in addition to three rural areas that were not included in the study. The regional climate is classified as humid subtropical mesothermal, characterised by hot, rainy summers and mild to cold winters (Peel et al., 2007). The mean annual temperature is 22.4°C, and the mean annual precipitation is 1867 mm (Climate‐Data, 2024). Foz do Iguaçu has been an endemic region for dengue since the 1990s, experiencing recurrent outbreaks and cyclical epidemic transmission patterns every 4–5 years (Leandro, Castro, et al., 2024). Furthermore, the municipality maintains standardised entomological and meteorological datasets essential for this investigation.

For the purpose of inspection standardisation, properties were defined as individual residential or commercial units, following criteria established by the Brazilian Ministry of Health. Apartment buildings were excluded, as vector surveillance and control activities are conducted solely in ground‐level common areas of condominium complexes.

### Data collection and organization

Entomological data were obtained from the Zoonosis Control Center (CCZ) of Foz do Iguaçu. Data collection relied on two primary methodologies: the Rapid Index Survey for *A. aegypti* (LIRAa) and the capture of adult mosquitoes using Adultraps (Leandro, Ayala, et al., 2022 and Leandro, de Castro, et al., 2022). LIRAa is a standardised entomological surveillance approach employed in Brazil to estimate larval infestation levels of *A. aegypti* in urban settings (Brasil, 2009; Coelho et al., 2008). It employs a two‐stage random sampling procedure, where blocks of houses serve as the primary sampling unit, and individual residences within these blocks are the secondary sampling unit. A relatively small subset of households, typically up to 450 out of a total of 9000–12,000 per district, is sampled 4–6 times annually. During larval surveys, health agents identify potential breeding sites of *A. aegypti* in containers located within the dwellings in which the householder granted verbal consent for inspection. Therefore, artificial and natural containers are inspected, and if any mosquito larvae are found, they are collected for morphological identification using local taxonomic keys and a stereo microscope at a local entomology laboratory facility (Leandro, Ayala, et al., 2022 and Leandro, de Castro, et al., 2022).

The Adultrap is specifically engineered to capture gravid *A. aegypti* females as they seek oviposition sites. Its primary attractant is water, which is stored in an inaccessible reservoir at the base of the device to prevent egg deposition. Females enter the trap through a wide opening at the top and are subsequently confined within an internal chamber that restricts their escape (Gomes et al., 2007; Maciel‐de‐Freitas et al., 2008). A total of 3476 Adultraps were installed across the Foz do Iguaçu city, positioned in peridomestic environments at a ratio of one trap per 25 properties. Therefore, the spatial distribution of Adultraps was uneven, with more traps allocated in the areas of the city with higher human density. These traps were inspected bimonthly, specifically during the first four days of odd‐numbered months. All captured mosquitoes were removed, transported to the laboratory for taxonomic identification and counting (Leandro, Ayala, et al., 2022 and Leandro, de Castro, et al., 2022).

Data collection spanned from January 2017 to November 2021, with bimonthly sampling. To produce the infestation indices for immature and adult *A. aegypti* stages, bimonthly inspections were carried out six times a year for 5 years, covering 100% of the municipality's urban area. Each two‐month period, 4500 properties were inspected to identify and classify breeding sites for *A. aegypti* larvae and pupae, totalling 135,000 properties inspected during this period. A fraction of those properties (3476) had Adultraps that were inspected every two months, resulting in approximately 105,000 visits. Meteorological data for the same period were obtained from the Paraná Meteorological System (SIMEPAR), with monthly records of accumulated precipitation and mean temperature on a point‐specific basis. Data standardisation procedures were implemented to ensure consistency and comparability across all analyses. Climatic data used in the analysis are available elsewhere (Freitas et al., 2026).

### Entomological indices

*Aedes aegypti* infestation was assessed using four entomological indices that estimate the positivity of breeding sites and the density of adult mosquitoes while considering their relationship with meteorological variables. Among the traditional LIRAa indicators, the HI and the BI were employed. These indices represent, respectively, the proportion of properties containing larvae or pupae and the number of positive breeding sites per 100 inspected properties, respectively (Focks & Chadee, 1997).

For adult mosquito surveillance, data derived from Adultraps were used to calculate the Trap Positivity Index (TPI) and the Adult Density Index (ADI). The TPI denotes the percentage of positive traps among the total inspected, whereas the ADI corresponds to the ratio between the total number of captured mosquitoes, and the number of examined traps (Leandro, Ayala, et al., 2022 and Leandro, de Castro, et al., 2022).

### Statistical analysis

Each entomological index was evaluated using generalized linear mixed models (GLMMs), accounting for temporal pseudoreplication (Bates et al., 2015). In the models, climatic variables (monthly mean temperature and accumulated precipitation) were specified as fixed effects, whereas the response variables were the mean municipality‐wide values of the larval and adult indices (HI, BI, TPI and ADI). The random‐effect structure incorporated each resampling date as a continuous random factor, comprising 30 levels corresponding to each sampling event over time (Crawley, 2005).

A Gaussian distribution was assumed for the continuous response variables, given the nature of the indices, and models were implemented using the R package lme4. Significance values for fixed effects were obtained using the lmerTest package. Model selection was carried out using Akaikes information criterion (AIC), based on differences in corrected AIC values (ΔAICc) and Akaike weights (wAICc), which were used to classify and assess the relative support for each candidate model (Burnham & Anderson, 2002). ΔAICc was employed to compare AIC scores between the best‐fitting model and competing models, whereas Akaike weights quantified uncertainty in model selection by representing the probability that a given model is the best among the candidates, given the data (Burnham & Anderson, 2002; Richards et al., 2011).

The most strongly supported model was defined according to the following criteria: (1) rejection of the null GLMM hypothesis (*p* < 0.05), (2) lowest AIC value and (3) Akaike weight >0.7 (70% confidence set) (Burnham & Anderson, 2002). Models with ΔAICc <4.0 were considered statistically equivalent (Lukacs et al., 2007). ΔAICc and wAICc analyses were conducted using the R package bbmle.

## RESULTS

During the period from 2017 to 2021, the larval surveys conducted through the Rapid Survey of *A. aegypti* Infestation Indices (LIRAa) documented a substantial sampling effort in the municipality of Foz do Iguaçu. In total, 146,274 properties were inspected, of which 3793 were positive for *A. aegypti* larvae, yielding 4416 positive containers.

In the monitoring carried out with Adultrap traps, 73,127 trap observations were performed, with 7799 positive traps, resulting in the capture of 17,980 adult *A. aegypti* mosquitoes. Across the monitoring period, 11,962 adult *A. aegypti* were identified, of which 95.4% of sampled *A. aegypti* were females, providing a consistent indicator of adult vector abundance (Freitas et al., 2026).

Based on the climatic data obtained, the highest mean maximum temperature was recorded in March 2017, reaching 26.55°C, whereas the lowest mean minimum temperature occurred in September 2018, with an average of 15.7°C. The highest accumulated precipitation was observed in March 2021, totalling 676.6 mm, while the lowest value occurred in September 2019, with 45.6 mm (Figure 1).

**FIGURE 1:**
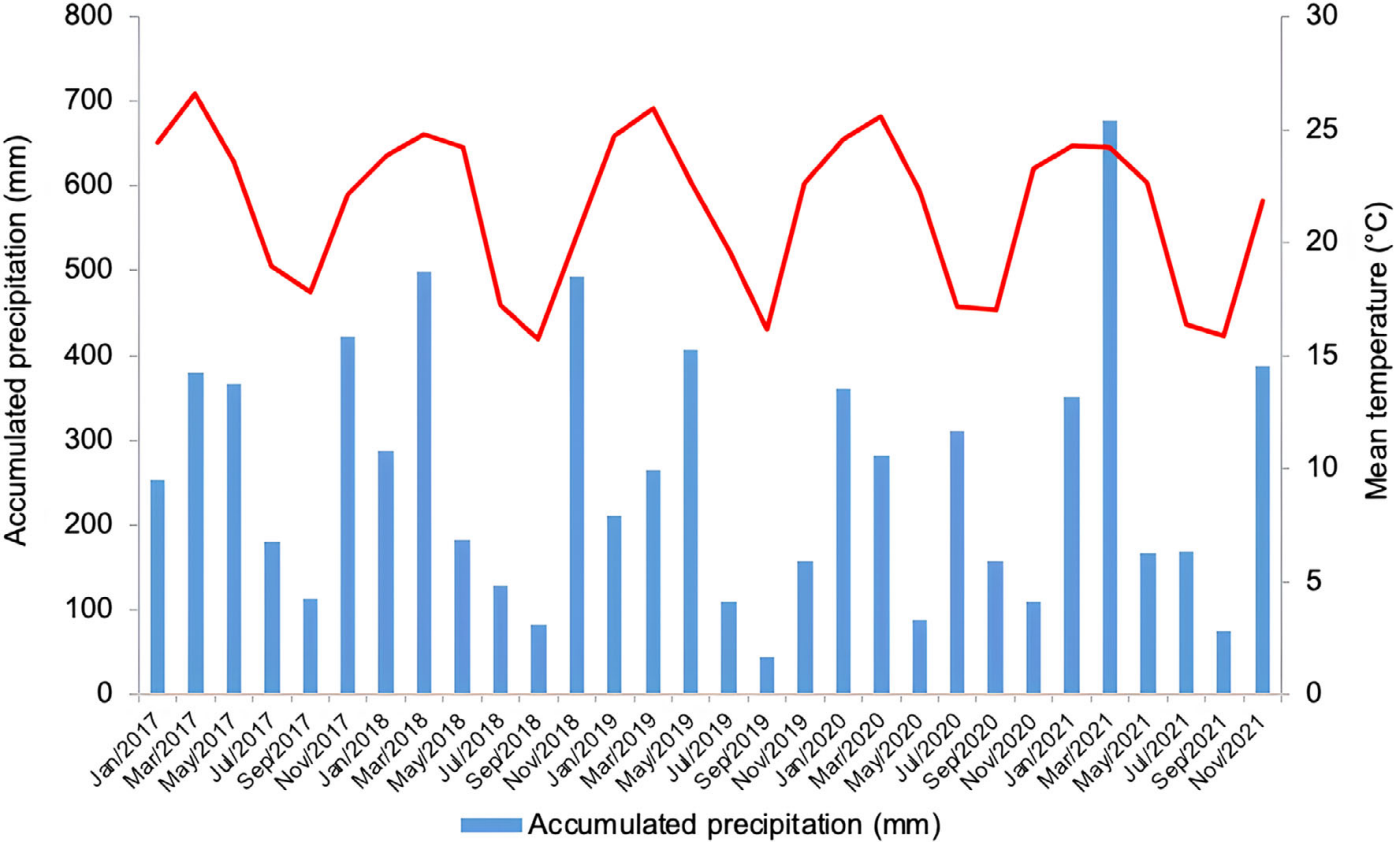
Monthly accumulated precipitation and mean temperature from 2017 to 2021 in Foz do Iguaçu, PR. *Source:* SIMEPAR (2025).

Regarding the entomological indicators, the (HI) reached its highest value of 5.70% in January 2021, and its lowest value of 0.50% in July 2020. The BI exhibited a maximum of 6.85% in January 2021 and a minimum of 0.64% in September 2019. That is, immature *A. aegypti* were found in every survey.

With respect to adult mosquito capture, the TPI reached its highest value in May 2021 at 19.6, and its lowest value in September 2018 at 3.5. The ADI showed its highest incidence in March 2021 at 72.44, and the lowest in September 2018 at 5.38 (Figure 2).

**FIGURE 2:**
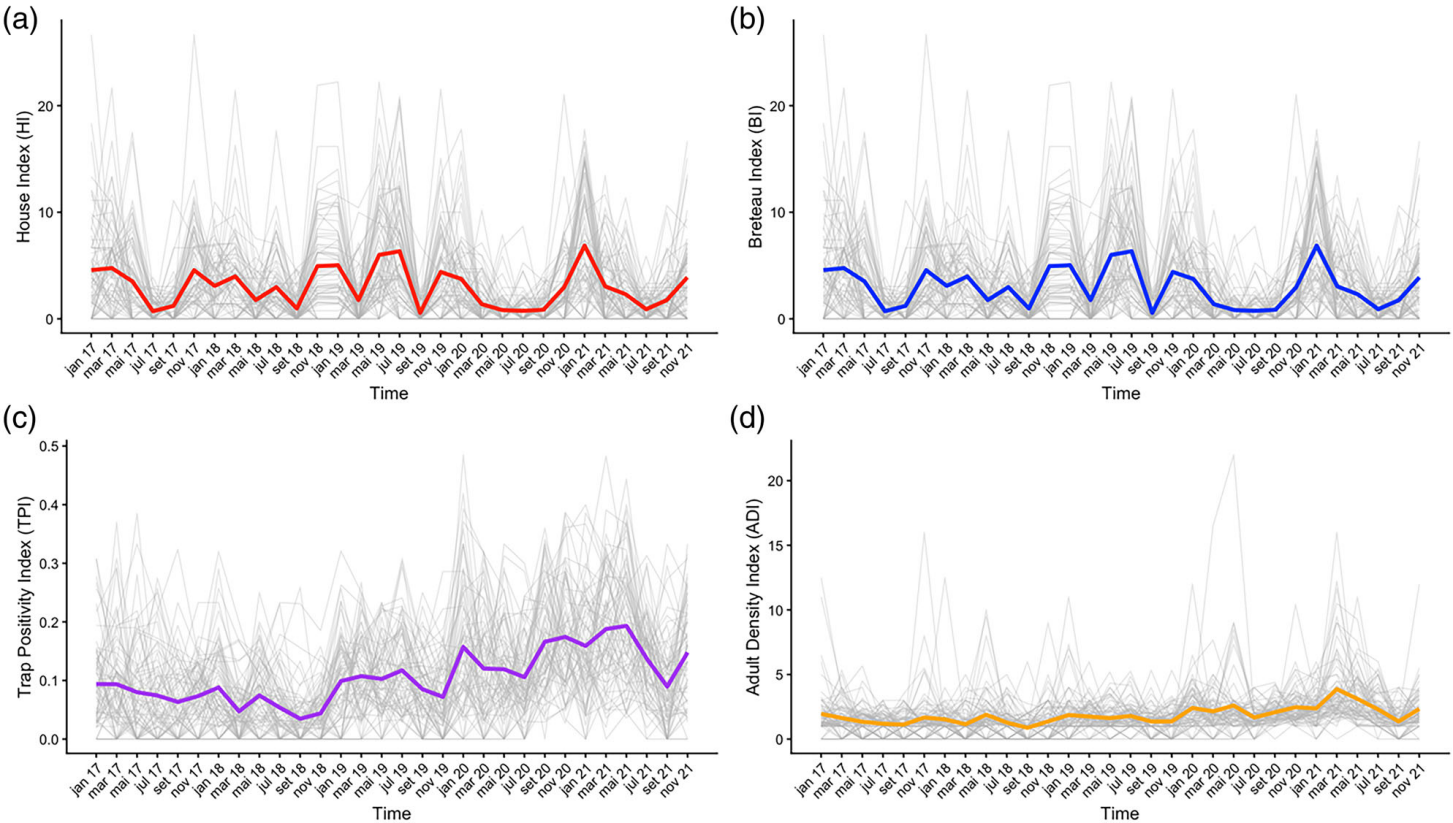
Entomological indices of *Aedes aegypti* from 2017 to 2021 in Foz do Iguaçu, PR. Each grey line represents the index variation in each of the 73 urban areas, whereas the coloured line indicates the corresponding average of each entomological indicator.

The larval entomological indices HI and BI, derived from *A. aegypti* immature forms, demonstrated statistically significant associations with the climatic variables analysed for Foz do Iguaçu from 2017 to 2021 (Tables 1 and 2). In contrast, the adult‐based indices TPI and ADI did not show significant relationships with the same climatic parameters. Consequently, larval indices proved more sensitive to the climatic factors assessed, indicating that increases in monthly mean temperature and monthly accumulated precipitation are associated with greater numbers of positive *A. aegypti* breeding sites. Moreover, among the interactions between larval indices and climatic factors, the association between the HI and mean temperature provided the strongest explanatory power (AIC, Table 3).

**TABLE 1:** Results of the generalized linear mixed models (GLMMs) assessing the relationship between accumulated precipitation and *Aedes aegypti* monitoring indices in Foz do Iguaçu, Brazil, from 2017 to 2021.

Index	Factor	Estimates	Std error	*t* value
HI	Intercept	1.398	0.540546	2.586*
	Precipitation	0.004	0.001654	2.783**
IB	Intercept	1.733075	0.677429	2.558*
	Precipitation	0.004937	0.002012	2.454*
IPA	Intercept	9.510244	1.522111	6.248*
	Precipitation	0.004093	0.005112	0.801
IDA	Intercept	16.60102	5.73379	2.895
	Precipitation	0.02523	0.01912	1.320

*Note*: Asterisks indicate significance levels: ** < 0.001, * < 0.05.

Abbreviations: ADI, Adult Density Index (Adultrap); BI, Breteau Index (LIRAa); HI, House Index (LIRAa); TPI, Trap Positivity Index (Adultrap).

**TABLE 2:** Results of the generalized linear mixed models (GLMMs) assessing the relationship between mean temperature and *Aedes aegypti* monitoring indices in Foz do Iguaçu, Brazil, from 2017 to 2021.

Index	Factor	Estimates	Std error	*t* value
HI	Intercept	−3.75501	1.40527	−2.672*
	Temperature	0.29407	0.06441	4.566***
IB	Intercept	−4.47949	1.81153	−2.473
	Temperature	0.34722	0.08302	4.182*
IPA	Intercept	1.7751	3.3333	0.533
	Temperature	0.4358	0.1531	2.847
IDA	Intercept	−7.6134	17.9468	−0.424
	Temperature	1.4871	0.8224	1.808

*Note*: Asterisks indicate significance levels: *** < 0.0001, * < 0.05.

Abbreviations: ADI, Adult Density Index (Adultrap); BI, Breteau Index (LIRAa); HI, House Index (LIRAa); TPI, Trap Positivity Index (Adultrap).

**TABLE 3:** Results of the akaike information criterion (AIC) ranking the most parsimonious models (in descending order) predicting the relationship between climatic factors and *Aedes aegypti* monitoring indices in Foz do Iguaçu, Brazil.*

Factor	ΔAICc	wAICc
HI × Temperature	0.0	0.987
HI × Precipitation	9.8	0.007
BI × Temperature	10.3	0.005
BI × Precipitation	20.3	<0.001

Abbreviations: ΔAICc, difference in the corrected Akaike information criterion; wAICc, corrected Akaike information criterion weights; BI, Breteau Index (LIRAa); HI, House Index (LIRAa).

* Based on *p* ≤ 0.05 in generalized linear mixed models (GLMMs). Each model represents the relationship between individual climatic factors (mean temperature and accumulated precipitation) and mosquito monitoring indices.

## DISCUSSION

This study aimed to elucidate the relationship between climatic variables and both immature (HI and BI) and adult (TPI and ADI) entomological indices of *A. aegypti*. The findings demonstrate that indices based on larval sampling exhibited measurable susceptibility to fluctuations in temperature and precipitation. Both HI and BI were responsive to thermal variation, highlighting their relevance for entomological surveillance; however, their robustness is intrinsically dependent upon methodological standardisation and the operational rigor of field surveillance to ensure representative and reliable data (Codeço et al., 2015). Larval infestation displayed pronounced seasonality, strongly modulated by temperature and precipitation patterns, consistent with the ectothermic physiology of the vector, whose biological processes are directly governed by environmental conditions (Heinisch et al., 2019; Moser et al., 2023). Temperature exerts a pivotal influence on metabolic activity and developmental rates of immature stages, thereby accelerating the life cycle and intensifying oviposition activity under warmer conditions (Gimenez et al., 2020). Empirical evidence from other endemic sites in Brazil corroborates this relationship, demonstrating increased larval abundance during periods of elevated temperature, wherein accelerated development occurs despite the potential for density‐dependent mortality at higher population levels (Marinho et al., 2016). Notwithstanding such constraints, temperature remains a decisive factor in reducing the time required for adult emergence.

Notably, the present findings reveal a somewhat unexpected pattern, wherein larval‐based indices exhibited stronger and more consistent associations with climatic variables than adult‐based metrics. This observation appears to diverge from a growing body of literature that advocates for the superiority of adult surveillance systems in the development of early‐warning frameworks for arboviral transmission, based on the premise that adult abundance more directly reflects the epidemiologically relevant segment of the vector population (Codeço et al., 2015; Jurado‐Sánchez et al., 2025; Leandro, Ayala, et al., 2022; Leandro, de Castro, et al., 2022; Leandro, Oliveira, et al., 2024; Shahar et al., 2022). Indeed, adult‐based indices have been increasingly promoted as more sensitive indicators of transmission risk, particularly when coupled with real‐time trapping technologies and predictive modelling approaches (Njaime et al. 2024; González‐Pérez et al. 2022; Silva et al. 2025). However, the results presented here suggest that, at least within the studied context, immature‐stage indicators may provide a more robust signal of environmental forcing, likely due to their more immediate and direct dependence on climatic conditions. This apparent discrepancy underscores the need for a more nuanced interpretation of surveillance metrics, recognising that the relative performance of larval versus adult indices may be context‐dependent and mediated by local ecological, infrastructural and operational factors. Consequently, rather than representing a contradiction, these findings may indicate that larval indices retain substantial value within integrated early‐warning systems, particularly in settings where adult surveillance is constrained by methodological limitations or environmental heterogeneity.

Precipitation similarly exerted a significant effect on infestation indexes based on larval sampling, corroborating prior studies that identify rainfall as a key driver of breeding‐site availability and egg hatching dynamics (Miyazaki et al., 2009; Rivas et al., 2018; Wilke et al., 2017). However, this relationship is inherently non‐linear, as precipitation alone is insufficient to sustain population growth under suboptimal thermal conditions (Heinisch et al., 2019). Furthermore, intense rainfall events may lead to the flushing of temporary breeding sites, resulting in the displacement or mortality of larvae, thereby likely reducing local populations (Codeço et al., 2015). The comparatively stronger influence of temperature observed in this study may also reflect the characteristics of urban environments, which harbour persistent artificial breeding sites, including uncovered water reservoirs, discarded tyres, drainage systems and improperly managed containers, thereby sustaining vector populations even during periods of limited rainfall (Benitez et al., 2021). Additionally, the remarkable desiccation resistance of *A. aegypti* eggs enables prolonged viability under dry conditions, facilitating rapid increases in larval density following subsequent precipitation events (Costa et al., 2022).

In contrast, adult indices exhibited no statistically significant association with climatic variables, underscoring the complexity of ecological and behavioural processes governing adult mosquito dynamics. Adult mosquitoes are capable of exploiting microhabitats characterised by greater environmental stability, thereby reducing their exposure to macroclimatic variability and influencing their detectability in trapping systems (Erraguntla et al., 2021; Johnson et al., 2015; Terradas et al., 2024). While predictive models frequently incorporate macroclimatic parameters, such approaches often fail to capture the thermal heterogeneity and structural complexity of urban landscapes, where shaded and humid microenvironments provide relatively stable conditions that influence adult survival, dispersal and activity patterns (Erraguntla et al., 2021). Moreover, additional determinants, including insecticide application, interspecific interactions, urban spatial configuration and broader‐scale processes such as climate change, contribute to variation in adult abundance and detectability (Schrama et al., 2018). These multifactorial influences likely account for the absence of a statistically significant relationship between climatic variables and adult indices observed herein.

The use of entomological indices to monitor seasonal fluctuations in mosquito population density constitutes a central component of vector surveillance (Codeço et al., 2015). It should be recognised, however, that all indices have inherent strengths and limitations, including the four entomological indicators adopted in the present study. Larval‐based indices, for instance, are widely used and operationally convenient, but they are often poorly correlated with adult mosquito abundance and, consequently, with the temporal dynamics of dengue incidence. Moreover, a critical dimension of entomological surveillance is frequently overlooked: container productivity (Tun‐Lin et al., 1995). In many routine surveillance programmes, larval sampling involves collecting only a fraction of the immature stages present in breeding sites, primarily for subsequent species identification. Because larvae and pupae are not exhaustively quantified, estimates of container productivity are often unavailable (Focks, 2003). This limitation constrains the interpretation of spatial and temporal patterns of infestation, particularly in settings where a small number of highly productive containers may contribute disproportionately to adult mosquito emergence. Adult‐based indices, in contrast, may provide a closer approximation of epidemiologically relevant vector abundance and, in some contexts, show stronger associations with dengue transmission. Nevertheless, these approaches also present important methodological and interpretative challenges. For several adult trapping systems, the trap density required to accurately capture fine‐scale spatial and temporal heterogeneity in *A. aegypti* abundance remains insufficiently defined. Similarly, the relationship between trap catch numbers and actual transmission risk is still not fully established. Mosquito abundance is a fundamental component of vectorial capacity and is more directly estimated through adult trapping than through larval surveys. This is because adult traps allow the enumeration and taxonomic identification of the epidemiologically relevant life stage more reliably than immature‐stage surveys. Thus, while no single entomological index can fully characterise infestation or transmission potential, adult‐based surveillance may offer a more informative measure of vector abundance when the objective is to approximate the mosquito population component most directly linked to arbovirus transmission.

The patterns identified in this study are also consistent with the historical emphasis of vector‐control strategies in Brazil, which have predominantly focused on household‐level interventions and the elimination of domestic breeding sites. Although such measures are indispensable, they may inadvertently neglect highly productive breeding sites located in public and structural environments. Previous investigations have identified storm drains, catch basins and non‐disposable containers as epidemiologically significant habitats (Paploski et al., 2016). In Mexico, a comprehensive survey revealed the presence of residual water in all inspected storm drains, of which 60% harboured *A. aegypti* larvae or adults, whereas household inspections identified only a single positive container. The authors estimated that storm drains contributed approximately 12 adult mosquitoes per day, whereas residential containers contributed none (Manrique‐Saide et al., 2013). Such findings underscore the importance of considering complex urban infrastructures as key components of vector ecology that are frequently overlooked by conventional control strategies.

The lack of correlation between climatic variables and adult indices is further supported by recent findings in the same municipality. In Foz do Iguaçu, Leandro, Castro, et al. (2024) evaluated the association between *A. aegypti* abundance and dengue incidence in children over a 13‐month period during the COVID‐19 pandemic and reported no relationship between adult‐based entomological indices and disease occurrence. Notably, environmental variables such as temperature and precipitation also exhibited no direct association with dengue incidence, suggesting that social and ecological determinants—including human mobility, population immunity, urban infrastructure and behavioural practices—play a more influential role in transmission dynamics. The authors further emphasised that trap performance may not accurately reflect true mosquito abundance, as capture rates are influenced by factors such as proximity to breeding sites and water storage practices.

Collectively, the present findings demonstrate that accumulated precipitation and mean temperature significantly influence *A. aegypti* indices based on larval sampling, while exerting no detectable effect on adult indices. These results reinforce the primacy of local‐scale determinants, including urban microclimates, the availability of alternative breeding sites and environmental heterogeneity in shaping vector population dynamics. Among the indices evaluated, HI exhibited greater sensitivity to climatic variation than the BI, whose comparatively attenuated response may reflect limitations in capturing the spatial heterogeneity of infestation. Conversely, adult indices derived from Adultrap showed no significant association with climatic variables, likely reflecting the influence of unmeasured ecological and operational factors, although these traps remain valuable due to their operational simplicity, low cost and practical applicability. The findings further corroborate the responsiveness of LIRAa to climatic variability and highlight the necessity of expanding vector‐control strategies beyond residential environments to encompass both conventional and non‐conventional breeding sites, including urban infrastructure such as stormwater drainage systems. Effective dengue control, therefore, requires integrative approaches that incorporate climatic, ecological, social and structural determinants. Future research should prioritise the investigation of urban microclimates and additional ecological drivers to enhance the accuracy of vector surveillance and the efficacy of control interventions.

## AUTHOR CONTRIBUTIONS

**Angie Nataly Aire Laureano:** Investigation; methodology; writing – original draft; writing – review and editing; conceptualization. **André de Souza Leandro:** Conceptualization; investigation; validation; methodology; data curation; writing – original draft; writing – review and editing. **Rafael Maciel de Freitas:** Conceptualization; investigation; funding acquisition; writing – original draft; writing – review and editing; formal analysis; visualization. **Wagner Antonio Chiba de Castro:** Conceptualization; investigation; writing – original draft; writing – review and editing; methodology; formal analysis; data curation; supervision.

## CONFLICT OF INTEREST STATEMENT

The authors declare that they have no competing interests.

## Data Availability

The raw data supporting the findings of this study are available in the citation Freitas et al. (2026) at https://doi.org/10.6084/m9.figshare.32429847.
